# Interior Profile Accuracy Assessment Method of Deep-Hole Parts Based on Servo Drive System

**DOI:** 10.3390/s24206554

**Published:** 2024-10-11

**Authors:** Jintao Liang, Kaixin Wang, Xiaotian Song, Xiaolan Han

**Affiliations:** 1 School of Mechano-Electronic Engineering, Xidian University, Xi’an 710126, China; 2 College of Mechanical Engineering, Xi’an Shiyou University, Xi’an 710065, China

**Keywords:** deep-hole, accuracy assessment, servo drive, least square method (LSM), gradient descent algorithm (GDM)

## Abstract

Dimensional and profile measurements of deep-hole parts are key processes both in manufacturing and product lifecycle management. Due to the particularity of the space conditions of deep-hole parts, the existing measurement instruments and methods exhibit some limitations. Based on the multi-axis, highly precise servo drive system, a novel measuring device is developed. The laser displacement sensors are fed by the flux-switching permanent magnet linear motor, and the part is rotated by the servo motor. On this basis, the assessment methods of roundness, straightness, and cylindricity are proposed by employing the least square method (LSM). Additionally, considering the axial center deviation between the sensors and the part, the rotating center coordinate is optimized by the gradient descent algorithm (GDM). Then, the measurement system is constructed and the experiment study is conducted. The results indicate favorable evaluation error of the LSM fitting and GDM iteration. Compared with the coordinate measuring machine (CMM), the measured results show good consistency. In the error analysis, the angle positioning error of measured point is less than 0.01°, and the axial positioning error is less than 0.05 mm. The proposed system and assessment method are regarded as a feasible and promising solution for deep-hole part measurements.

## 1. Introduction

Parts with a depth-to-diameter ratio (L/D) greater than five are typically defined as deep-hole parts, which are widely used in aviation, aerospace, military weapons, energy exploration, etc. [1,2]. As the demands for improved performance, reduced weight, and increased efficiency increase in manufacturing various equipment, more deep-hole parts with larger L/D ratios are emerging. The larger the L/D ratio, the higher the requirements for each machining process needed. The measurement of the dimensions and profile accuracy (diameter, straightness, roundness, cylindricity, etc.) of deep holes is a crucial issue. Not only can these measurements evaluate the machining quality but they also can be used for a maintenance analysis. They provide a reliable basis for improving the machining accuracy of deep-hole parts and optimizing process parameters [3,4]. Contrary to the demand for high-precision deep-hole parts, the current deep-hole measurement generally remains at the manual measurement stage. Methods such as gauge measurements, stress–strain measurements, and lever measurements are used to detect the dimensions and interior profile accuracy of deep holes, but these methods are often limited by the large depth-to-diameter ratio and narrow internal space inside the deep hole, and the measurement accuracy is low. High-precision equipment like a coordinate measuring machine (CMM) and laser trackers is expensive and complex to operate, and it is difficult to promote widely across the whole industry. Therefore, high-precision, cost-effective, automated, and intelligent deep-hole measuring equipment and assessment methods should be urgently developed [5,6].

Many studies have been conducted to measure various deep-hole parts. Ma et al. [7] introduced a pinhole diameter measuring device using a cylindrical capacitive probe, and reliable results from *ϕ* 1.85 mm to *ϕ* 7 mm in ≤18 mm depth holes can be achieved. Elfurjani et al. [8] presented a wire-based rotating probe and an acoustic emission touch detection system, measured micro-scale holes of less than 1.0 mm in diameter and 10 mm in depth, and calculated the roundness. Arunkumara et al. [9] investigated the effects of the deep-hole drilling parameters on the quality of the hole produced, a 10 mm diameter and 120 mm deep hole were considered, and the circularity and cylindricity were measured using CMM. Shi et al. [10] developed a “DN800 diameter detector” for detecting a pipeline in both the radial and circumferential directions, the radial measurement error was less than ±2 mm, and the dip angle accuracy was within ±0.5°. Bian et al. [11] constructed a hole diameter measuring machine based on spherical scattering electrical field probing; the sensor resolution was 1 nm, and 0.2 mm uncertainty with a 20 mm diameter was achieved. Katsuki et al. [12] developed a laser-guided-type deep small-sized hole measurement system, with a 17–21 mm diameter and 1000 mm length. The measured hole deviation and roundness profile were discussed. Song et al. [13] proposed a multi-sensor integrated measurement system for deep-hole parts with a diameter of 150–165 mm and a length of 600–20000 mm. the roundness and axis straightness errors were less than 10 μm, and the repeatability accuracy in shape error was less than 1 μm. Zhao et al. [14] presented a deep-hole measurement instrument based on a laser and monocular camera, and the diameter of deep hole was up to 300 mm. Additionally, the repeatability error was improved to 0.012 mm using perspective transformation to compensate for sensor thermal errors. Due to the special spatial conditions of deep holes, these presented measuring method have certain limitations. When the hole diameter is small, only a simple probe or sensor can be stretched into the narrow inside space, but the stretching depth is restricted and the reference coordinate error of the external support is unknown. When the hole diameter is large enough, although the measuring device can be operated along the hole axis unlimitedly, the moving position and measured dimensions should be detected by multiple sensors, which increase the complexity and cost of the device. Therefore, so far, no unified method has been widely applied in actual manufacture.

Referring to previous research, employing a displacement sensor to scan the interior profile of deep-hole parts point by point is considered a simple and feasible measurement method. However, significant measurement errors would be caused by the reference and positioning errors of the scanning mechanism. Based on a high-precision servo drive system, a deep-hole measuring device is constructed in this paper. Two complementary laser displacement sensors are driven by a flux-switching permanent magnet linear motor (FSPMLM) for axial feeding in the deep hole, while the deep-hole part is rotated by an AC servo motor. According to the least squares method (LSM), the roundness, straightness, and cylindricity errors are evaluated from the measured points at equal angular intervals on a series of circular sections with equal displacement intervals. Considering the positional offset among the source center of the laser displacement sensor, the axial center, and the rotating center of the part, the gradient descent method (GDM) is employed to eliminate the measurement errors.

## 2. Measuring Device Based on a Servo Drive System

### 2.1. Device Design

The deep-hole measurement can be classified into contact and non-contact types. Compared with contact measurement, the non-contact type avoids an effect on the metal interior surface and provides a wider measurement range. So, the non-contact type laser displacement sensor is employed. To obtain the point cloud of the interior profile of a deep hole, a scanning mechanism is required to realize both the rotating motion in any cross-section and the axial feeding motion for the cross-section position.

**Figure 1:**
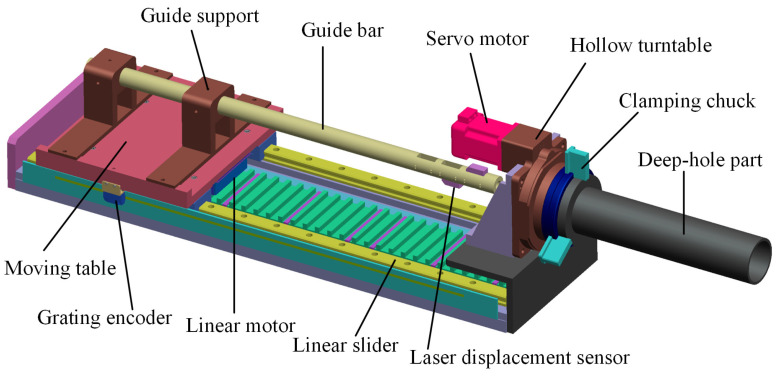
Design of the deep-hole measuring device based on a servo drive system.

In recent years, our research group focused on novel servo drive systems such as FSPMLM, through which precise dynamic tracking and position control can be achieved [15,16]. At present, we are committed to applying the servo drive system to construct deep-hole machining and measuring equipment. According to the above laser scanning requirement, an accurate deep-hole interior profile measuring device is proposed, as shown in Figure 1. Based on the previously developed FSPMLM feed apparatus [15], a guide bar is arranged on a moving table. For complementary measurements, two laser displacement sensors are symmetrically installed at the front end of the guide bar. A hollow turntable driven by an AC servo motor is set at the axial end of the FSPMLM stator base. A clamping chuck, which is used to coaxially hold various deep-hole parts with different diameters, is coaxially connected to the hollow turntable.

Combined with the grating encoder, high-precision, closed-loop control of any feeding position of the laser displacement sensor on the guide bar can be achieved by the FSPMLM. A high-precision rotary encoder is configured in the servo motor for closed-loop control, with which the deep-hole part on the hollow turntable can be driven to rotate around the center axis at any angle setting. Therefore, with the laser displacement sensor feeding axially into the measured deep-hole part at a given position, the distance from the laser source to a point on the inner profile can be measured. Additionally, with the deep-hole part rotating circumferentially, the measured distances of the points on the same circular section can be obtained. That is to say, through the 2-axis linear-rotating servo drive system, any positioning point on the interior profile of the deep hole can be detected so that the interior profile can be reconstructed through the point cloud data, and then dimension and accuracy parameters, such as the inner diameter, roundness, and straightness, can be calculated.

### 2.2. System Construction

**Figure 2:**
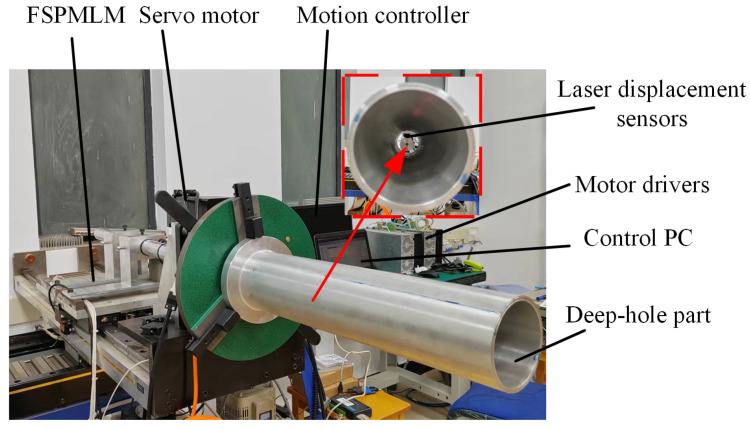
Prototype of the servo drive measuring device.

Based on the existing FSPMLM feed apparatus, a deep-hole measuring device is set up, as shown in Figure 2. The rated speed of the FSPMLM is 0.5 m/s, the maximum speed is 1.5 m/s, the rated thrust is 500 N, and the maximum feed stroke is 600 mm. The resolution of the grating encoder is 1 μm. Since this apparatus prototype was originally used for the optimization of the design and verification of the FSPMLM, its feeding stroke is relatively short. In practical applications, the feeding stroke of the FSPMLM can be extended by lengthening the stator and its base according to the requirement of measurable axial lengths of deep-hole parts. It should be noted that the stator of the designed FSPMLM exhibits a salient pole core structure [17]. The cost increase for extending the FSPMLM stroke is minimal. Therefore, theoretically, the FSPMLM can achieve measurements of any hole depth.

In addition, a servo motor with rated power of 400 W and rated speed of 3000 rpm is selected to drive the deep-hole part rotation, and a 23-bit single-turn absolute encoder is equipped in the motor. The reduction ratio of the hollow turntable is 10, and the installation diameter of the turntable is 130 mm. A self-centering, three-jaw chuck is used, the maximum outer diameter of the parts that can be clamped by the chuck is 360 mm, and the self-centering accuracy of the chuck is 0.05–0.15 mm. Two laser displacement sensors with a 1 μm resolution are used to measure the deep-hole part, the measurable inner diameter range of which is 80 mm–320 mm.

**Figure 3:**
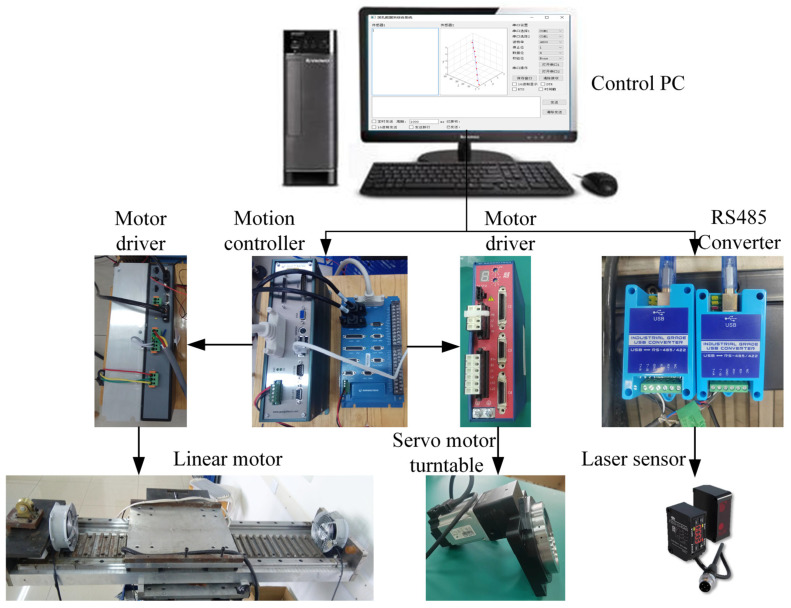
Construction of the servo drive measurement system.

The whole measurement system is constructed as shown in Figure 3. Measuring software V1 is programmed on a personal computer (PC) based on C#.net. The motion control instructions are sent from the measuring software V1 to the motion controller and then converted into the motor driver instructions for the FSPMLM and the servo motor. Through the motor driver for the FSPMLM, the feeding position of the laser displacement sensor can be closed-loop controlled. Meanwhile, the rotational position of the deep-hole part can be closed-loop controlled by the motor driver for the servo motor. This 2 degree-of-freedom (DOF) motion trajectory can be realized with the motion controller. On the other hand, based on the RS485 communication protocol, acquisition control instructions are sent from the measuring software V1 to the laser displacement sensors through the RS485-USB converter, and the measured data are received. Employing the interactive interface between C# and Matlab R2023a, the dimension and accuracy parameters are calculated through the Matlab R2023a program, and the results are displayed in the C# measuring software V1. Additionally, the measured data and calculated parameters are stored by the SQL Server database for further analysis.

### 2.3. Measurement Principle

Based on the proposed measurement system, there are two scanning and measuring methods for the interior profile of deep-hole parts.

First method: The two laser distance sensors are driven by the FSPMLM from one end of the deep-hole part. At regular intervals, the distance to the contour points is measured until reaching the other end of the deep hole or the bottom of the blind hole. Thence, the position information of the discrete contour points along the upper and lower generators are captured to construct an axial cross-section, from which the inner diameter, central axis, and straightness error can be calculated. After the laser distance sensors are driven back to the initial position, the workpiece is rotated with a certain angle by the servo motor, and the above measurement processes are repeated to measure another two generators. Repeating the rotation of the workpiece with the same interval angle, the point clouds with equal angular intervals on a series of circular cross-sections with equal axial interval are obtained.

Second method: From the one end of the deep-hole part, the part is rotated at an equal angular interval, and the discrete points are measured by the laser displacement sensor on the same circular cross-section. Then, by feeding the same interval displacement by the FSPMLM, the interval circular cross-section points are measured repeatedly until reaching the other end of the deep hole. Thus, the point cloud information of the interior profile can be obtained.

**Figure 4:**
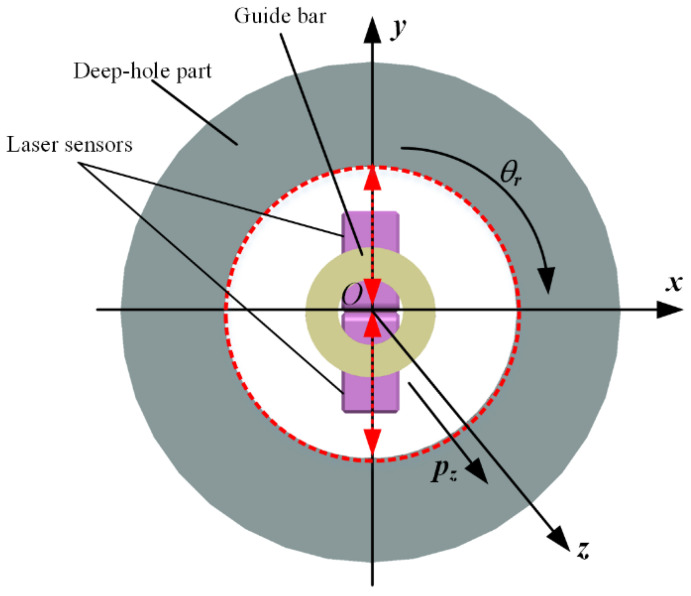
Measurement principle of the proposed system.

In an ideal condition, the guide bar, the part, and the part rotation are on the same central axis, as shown in Figure 4. Dimension and profile parameters, such as the hole inner diameter, straightness error, and roundness error, can be calculated by one of the two methods.

## 3. Accuracy Assessment Method

**Figure 5:**
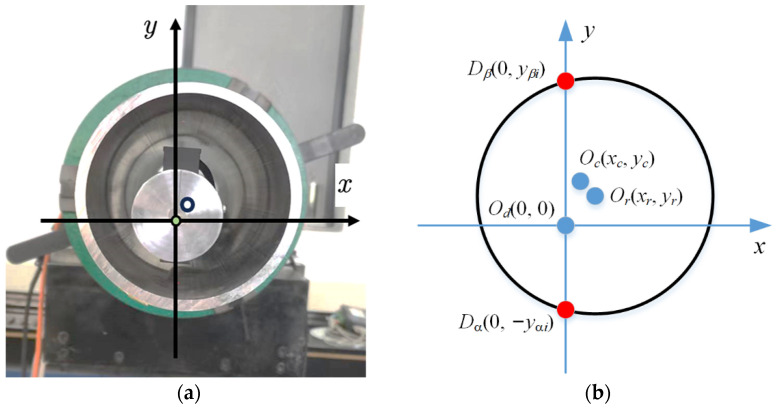
Deviation of the axial center. (**a**) actual image and (**b**) coordinates established.

In practice, due to the deviations existing in equipment manufacturing and chuck clamping, the axis of the guide bar, the axis of the turntable, and the axis of the deep-hole part cannot be completely aligned. This results in the center of the deep-hole cross-section (*O_c_*), the source of the laser distance sensor (*O_d_*), and the rotation center of the deep-hole part (*O_r_*) not coinciding at the same point, as shown in Figure 5. The central axis of the deep hole is not on the centerline of the axial cross-section formed by the upper and lower generators. Meanwhile, points on the circular cross-section of the deep hole are not on the same circumference after rotation. Therefore, various measurement uncertainty and errors exist. To avoid the influences of the manufacturing and positioning errors, methods for assessing the accuracy of roundness, straightness, and cylindricity are proposed.

### 3.1. Assessment of Roundness Accuracy

Firstly, the *x*–*y* coordinates are established using the source point of the laser displacement sensor *O_d_* as the original point (0, 0). Then, the coordinate of *O_c_* is defined as (*x_c_*, *y_c_*), and the coordinate of *O_r_* is (*x_r_*, *y_r_*), the values of which are unknown initially. For a one-time measurement, the coordinates of the hole cross-section points measured by the upper and lower laser sensors are *D_α_* (0, −*y_αi_*) and *D_β_* (0, *y_βi_*), respectively.

Taking the measured point of the upper laser sensor as an example, with the deep-hole part undergoing intervallic rotating *N* times in one cross-section, the *i*-th time measured point *D_α_* (0, −*y_αi_*) is rotated around the point *O_r_* (*x_r_*, *y_r_*) with the angle *θ_i_*, and *θ_i_* is (*N* − *i*)·2*π*. Then, the coordinates are transformed to a new value (*x_ni_*, *y_ni_*), and the mathematical representation is expressed as follows:

(1) $$\left\{\begin{array}{l} x_{ni}=(0-x_{r})\cdot \cos(\frac{N-i}{N}\cdot 2\pi )-(-y_{\alpha i}-y_{r})\cdot \sin(\frac{N-i}{N}\cdot 2\pi )+x_{r}\\ y_{ni}=(0-x_{r})\cdot \sin(\frac{N-i}{N}\cdot 2\pi )+(-y_{\alpha i}-y_{r})\cdot \cos(\frac{N-i}{N}\cdot 2\pi )+y_{r} \end{array}\right.,$$

**Figure 6:**
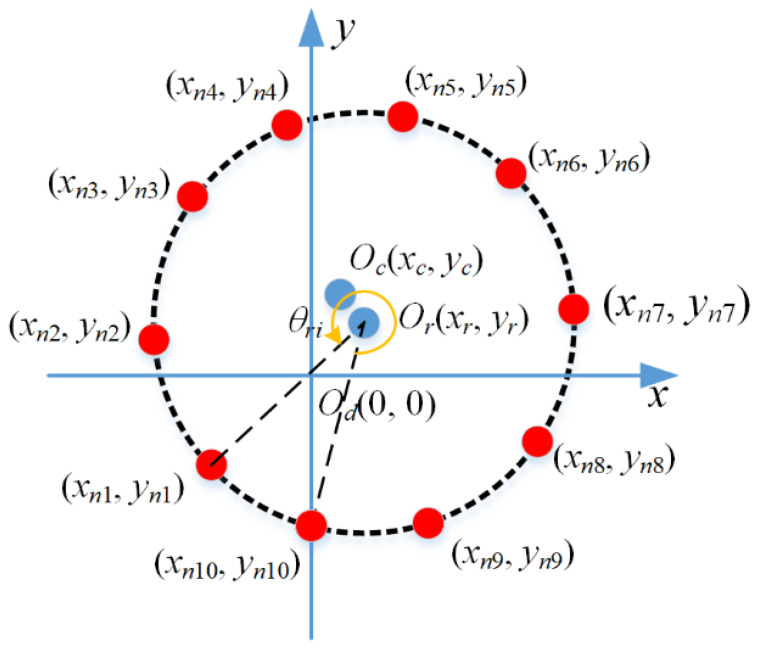
Coordinates of *N* measurement points on a circular cross-section.

Through the deep-hole part rotating a circle, the coordinates of *N* measured points (*x_ni_*, *y_ni_*), *i* = 1, 2, …, *N*, are obtained, as shown in Figure 6 (*N* = 10 for instance). Then, the center-point coordinate and the diameter of the circular cross-section can be calculated by fitting the *N* measured points based on the LSM.

The general equation of a circle is used as follows:

(2) $$x^{2}+y^{2}+a_{o}x+b_{o}y+c_{o}=0,$$

where

(3) $$\left\{\begin{array}{l} x_{c}=-0.5a_{o}\\ y_{c}=-0.5b_{o}\\ D_{o}=\sqrt{{a_{o}}^{2}+{b_{o}}^{2}-4c_{o}} \end{array}\right.,$$

*a_o_*, *b_o_*, and *c_o_* are the circle coefficients, (*x_c_*, *y_c_*) is the center-point of the circle, and *D_o_* is the diameter of the circle.

The *N* measured points are employed to fit the circle according to the LSM [18,19], and the objective function of LSM *F_o_* (*a_o_*, *b_o_*, *c_o_*) is established as follows:

(4) $$F_{o}(a_{o},b_{o},c_{o})=\sum_{i=1}^{N}{\left({x_{ni}}^{2}+{y_{ni}}^{2}+a_{o}x_{ni}+b_{o}y_{ni}+c_{o}\right)}^{2}$$

To gain the minimum value of *F_o_* (*a_o_*, *b_o_*, *c_o_*), the partial derivatives of *F_o_* with respect to *a_o_*, *b_o_*, and *c_o_* are calculated and set to zero, yielding the following equation:

(5) $$\left[\begin{array}{c} \sum_{i=1}^{N}{x_{ni}}^{3}+\sum_{i=1}^{N}x_{ni}{y_{ni}}^{2}\\ \sum_{i=1}^{N}{x_{ni}}^{2}y_{ni}+\sum_{i=1}^{N}{y_{ni}}^{3}\\ \sum_{i=1}^{N}{x_{ni}}^{2}+\sum_{i=1}^{N}{y_{ni}}^{2} \end{array}\right]+\left[\begin{array}{ccc} \sum_{i=1}^{N}{x_{ni}}^{2} & \sum_{i=1}^{N}x_{ni}y_{ni} & \sum_{i=1}^{N}x_{ni}\\ \sum_{i=1}^{N}x_{ni}y_{ni} & \sum_{i=1}^{N}{y_{ni}}^{2} & \sum_{i=1}^{N}y_{ni}\\ \sum_{i=1}^{N}x_{ni} & \sum_{i=1}^{N}y_{ni} & N \end{array}\right]\left[\begin{array}{c} a_{o}\\ b_{o}\\ c_{o} \end{array}\right]=\left[\begin{array}{c} 0\\ 0\\ 0 \end{array}\right],$$

Then, *a_o_*, *b_o_*, and *c_o_* are solved and the estimated values *x_c_*′, *y_c_*′, and *R_o_*′ are obtained by Equation (3).

The roundness error *f_o_* of the circular cross-section is defined as the difference between the maximum and minimum distances from each measured point to the center-point of the LSM fitting circle, which can be expressed as follows:

(6) $$f_{o}=\max_{i=1}^{N}\left(\sqrt{{\left(x_{ni}-{x_{c}}^{\prime }\right)}^{2}+{\left(y_{ni}-{y_{c}}^{\prime }\right)}^{2}}\right)-\min_{i=1}^{N}\left(\sqrt{{\left(x_{ni}-{x_{c}}^{\prime }\right)}^{2}+{\left(y_{ni}-{y_{c}}^{\prime }\right)}^{2}}\right),$$

However, considering that the coordinate of *O_r_* (*x_r_*, *y_r_*) is unknown, the coordinate of (*x_ni_*, *y_ni_*) cannot be solved only by Equation (1). In addition, the axis of the part and the axis of the turntable are not ideally collinear, which results in the differences between the coordinates of *O_r_*. Therefore, determining the unknown rotating center *O_r_* (*x_r_*, *y_r_*) is necessary for each measured circular cross-section.

With regard to a certain circular cross-section, two sets of measured points {*D_α_* (0, −*y_αi_*), *D_β_* (0, *y_βi_*), *i* = 1, 2, …, *N*} can be obtained by the upper and lower laser displacement sensors, respectively. According to Equation (1), with the same rotating center point (*x_r_*, *y_r_*), the coordinates of the two sets of circular cross-section points are transformed to {(*x_ni_^α^*, *y_ni_^α^*), (*x_ni_^β^*, *y_ni_^β^*), *i* = 1, 2, …, *N*}, respectively. Through the above LSM, the circle center coordinates (*x_c_^α^*′, *y_c_^α^*′) and (*x_c_^β^*′, *y_c_^β^*′) can be estimated such that

(7) $$\left\{\begin{array}{l} x_{c}^{\alpha \prime }\approx x_{c}^{\beta \prime }\\ y_{c}^{\alpha \prime }\approx y_{c}^{\beta \prime } \end{array}\right.$$

From Equations (1), (3) and (5), the functional relationships between *x_c_^α^*′ and *y_c_^α^*′, *x_c_^β^*′ and *y_c_^β^*′, and *x_r_* and *y_r_* can be established, respectively, and *x_r_* and *y_r_*_,_ can be optimized with initial values to satisfy Equation (7). An optimization objective function *F_a_*(*x_r_*, *y_r_*) can be established as follows:

(8) $$F_{a}(x_{r},y_{r})={\left[x_{c}^{\alpha \prime }(x_{r},y_{r})-x_{c}^{\beta \prime }(x_{r},y_{r})\right]}^{2}+{\left[y_{c}^{\alpha \prime }(x_{r},y_{r})-y_{c}^{\beta \prime }(x_{r},y_{r})\right]}^{2}$$

The GDM is applied for iterative optimization [20,21], and the gradients of *F_a_* $\left(\frac{\partial F_{a}}{\partial x_{r}},\frac{\partial F_{a}}{\partial y_{r}}\right)$ can be solved by the Matlab R2023a programming function, which is shown in Appendix A.

Setting (*x_r_*_0_, *y_r_*_0_) as the initial value, and the iterative learning rates are Δ*x_r_*, Δ*y_r_*, and Δ*x_r_* = Δ*y_r_*, and then the next iteration values are as follows:

(9) $$\left\{\begin{array}{l} x_{r1}=x_{r0}-{\left.\Delta x_{r}\cdot \frac{\partial F_{a}}{\partial x_{r}}\right|}_{x_{r0}}\\ y_{r1}=y_{r0}-{\left.\Delta y_{r}\cdot \frac{\partial F_{a}}{\partial y_{r}}\right|}_{y_{r0}} \end{array}\right.,$$

The above iterative calculation is repeated until *F_a_* is less than the set minimum value *δ*, and then the estimated values of the rotating center coordinates (*x_r_*′, *y_r_*′) are determined. On this basis, accuracy parameters, including the center, diameter, and roundness error, of the circular cross-section are calculated.

### 3.2. Assessment of Straightness Accuracy

The laser sensors are fed by the FSPMLM along the axial direction of the deep hole from one end of the deep-hole part with an interval displacement *l*_0_ after feeding *M* times to reach the end or the bottom of the part. So, the axial length of the hole *L_p_* is as follows:

*L_p_* = *l*_0_ × *M* (10)

*M* times of interval circular cross-section point measurements are conducted. Based on the above LSM and GDM, each center (*x_cj_*′, *y_cj_*′, *z_cj_*′) and diameter *D_oj_* are calculated, where {*z_cj_* = *l*_0_ × (*j* − 1), *j* = 1, 2, …, *M*.}. After connecting the centers of these *M* cross-sections, the central axis of the deep-hole part can be fitted by the LSM, and then the straightness error can be evaluated according to the general equation of a spatial straight line:

(11) $$\left\{\begin{array}{l} x=a_{l}z+b_{l}\\ y=c_{l}z+d_{l} \end{array}\right.,$$

where *a_l_*, *b_l_*, *c_l_*, and *d_l_*, are undetermined coefficients.

The axial line can be fitted by LSM with the *M* center points {(*x_cj_*′, *y_cj_*′, *z_cj_*′), *j* = 1, 2, …, *M*}, and the objective function of LSM *F_l_* (*a_l_*, *b_l_*, *c_l_*, *d_l_*) is established as follows:

(12) $$F_{l}(a_{l},b_{l},c_{l},d_{l})=\sum_{j=1}^{M}{\left({x_{ci}}^{\prime }-(a_{l}{z_{cj}}^{\prime }+b_{l})\right)}^{2}+{\left({y_{cj}}^{\prime }-(c_{l}{z_{cj}}^{\prime }+d_{l})\right)}^{2},$$

To gain the minimum value of *F_l_* (*a_l_*, *b_l_*, *c_l_*, *d_l_*), the partial derivatives of *F_l_* with respect to *a_l_*, *b_l_*, *c_l_*, and *d_l_* are calculated and set to zero, yielding

(13) $$\left\{\begin{array}{l} a_{l}=\frac{M\sum_{j=1}^{M}{x_{cj}}^{\prime }{z_{cj}}^{\prime }-\sum_{j=1}^{M}{x_{cj}}^{\prime }\cdot \sum_{j=1}^{M}{z_{cj}}^{\prime }}{M\sum_{j=1}^{M}{z_{cj}}^{\prime 2}-\sum_{j=1}^{M}{z_{cj}}^{\prime }\cdot \sum_{j=1}^{M}{z_{cj}}^{\prime }}\\ b_{l}=\frac{\sum_{j=1}^{M}{x_{cj}}^{\prime }-a_{l}\cdot \sum_{j=1}^{M}{z_{cj}}^{\prime }}{M}\\ c_{l}=\frac{M\sum_{j=1}^{M}{y_{cj}}^{\prime }{z_{cj}}^{\prime }-\sum_{j=1}^{M}y_{cj}\cdot \sum_{j=1}^{M}{z_{cj}}^{\prime }}{M\sum_{j=1}^{M}{z_{cj}}^{\prime 2}-\sum_{j=1}^{M}{z_{cj}}^{\prime }\cdot \sum_{j=1}^{M}{z_{cj}}^{\prime }}\\ d_{l}=\frac{\sum_{j=1}^{M}{y_{cj}}^{\prime }-c_{l}\cdot \sum_{j=1}^{M}{z_{cj}}^{\prime }}{M} \end{array}\right.,$$

Then, the center axis of the deep-hole part is evaluated from Equation (11).

An ideal cylinder is formed by the maximum distance from the *M* center points to the LSM fitting center axis, and the straightness error *f_l_* is determined as the diameter of the cylinder. To simplify the calculation, the deviation values from the measured points to the fitting axis are used to represent the distance values, i.e., *f_l_* can be expressed as follows:

(14) $$f_{l}=2\cdot \max_{j=1}^{M}\left(\sqrt{{\left({x_{cj}}^{\prime }-(a_{l}{z_{cj}}^{\prime }+b_{l})\right)}^{2}+{\left({y_{cj}}^{\prime }-(c_{l}{z_{cj}}^{\prime }+d_{l})\right)}^{2}}\right),$$

### 3.3. Assessment of Cylindricity Accuracy

The cylindricity error is generally defined as the minimum difference in the radius of the two coaxial cylinders that tightly contain all points on the actual cylindrical surface. In this paper, the above LSM fitting center axis is regarded as the axis of the coaxial cylinders. The distances from all (*N* × *M*) measured points to this fitting axis are calculated, and the cylindricity error *f_c_* is defined as the difference between the maximum and minimum values, as follows:

(15) $$\begin{array}{c} f_{c}=\max_{i=1}^{N}{,}_{j=1}^{M}\left(\sqrt{{\left(x_{nij}-(a_{l}{z_{cj}}^{\prime }+b_{l})\right)}^{2}+{\left(y_{nij}-(c_{l}{z_{cj}}^{\prime }+d_{l})\right)}^{2}}\right)-\\ \min_{i=1}^{N}{,}_{j=1}^{M}\left(\sqrt{{\left(x_{nij}-(a_{l}{z_{cj}}^{\prime }+b_{l})\right)}^{2}+{\left(y_{nij}-(c_{l}{z_{cj}}^{\prime }+d_{l})\right)}^{2}}\right) \end{array}$$

## 4. Experiment

**Figure 7:**
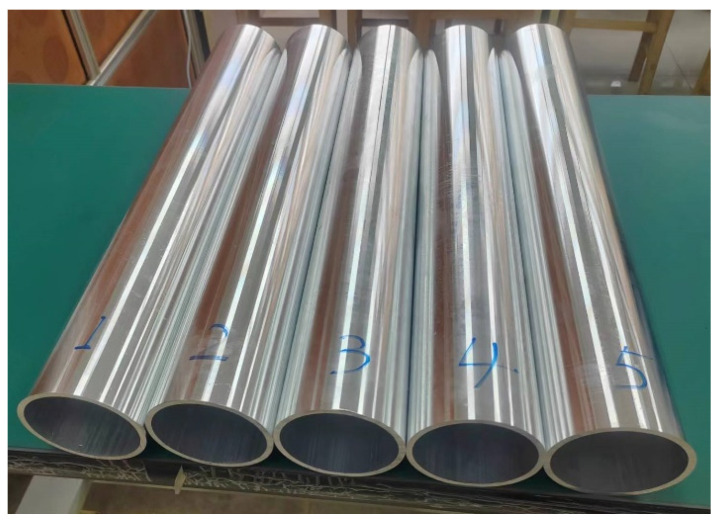
Five aluminum alloy deep-hole parts from a certain batch.

An experimental study was conducted to verify the effectiveness and performance of the proposed servo drive measurement system and accuracy assessment method. Five aluminum alloy deep-hole parts from a certain batch were supplied to measure, as shown in Figure 7. The hole diameters were machined to 89 mm, and the hole axis lengths were 500 mm. The laser displacement sensors were fed to the hole port first, and the part was driven to rotate 36° to conduct one measurement., i.e., *N* = 10 measured points were collected on a circular cross-section. The FSPMLM feeding interval was set to 60 mm, and then *M* = 9 measured cross-section points were obtained. At each measured point, the displacement was detected by the laser sensor three times to eliminate the external disturbance. Furthermore, the measurement process of each part was conducted three times.

Using the deep-hole part No. 1, as illustrated, the eighth circular cross-section was discussed and the displacement values of 10 measured-points were acquired by the upper and lower laser displacement sensors, as shown in Table 1.

**Figure 8:**
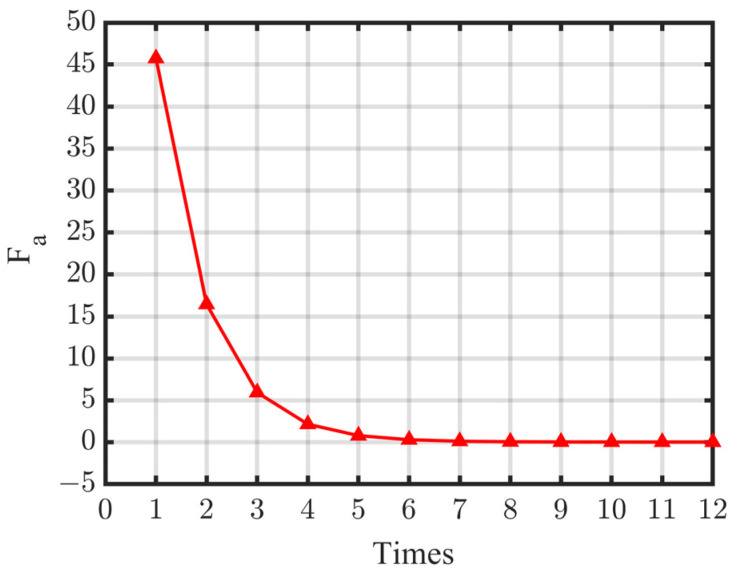
Iterative calculation of the values of *F_a_*.

Employing the proposed assessment method, the coordinates of the rotating center of the deep-hole part was iteratively optimized by GDM. The initial value of *O_r_* (*x_r_*, *y_r_*) was set to (0, 0), the iterative learning rate was Δ*x_r_* = Δ*y_r_* = 0.1, the maximum number of iterations was 100, and the target function *F_a_* value for stopping the iteration was 0.001. The variation of the target function through iterative calculation is shown in Figure 8. It can be seen that after 12 iterations, *F_a_* = 0.0006, which is less than the set target. Then, *O_r_* (*x_r_*, *y_r_*) can be determined.

**Figure 9:**
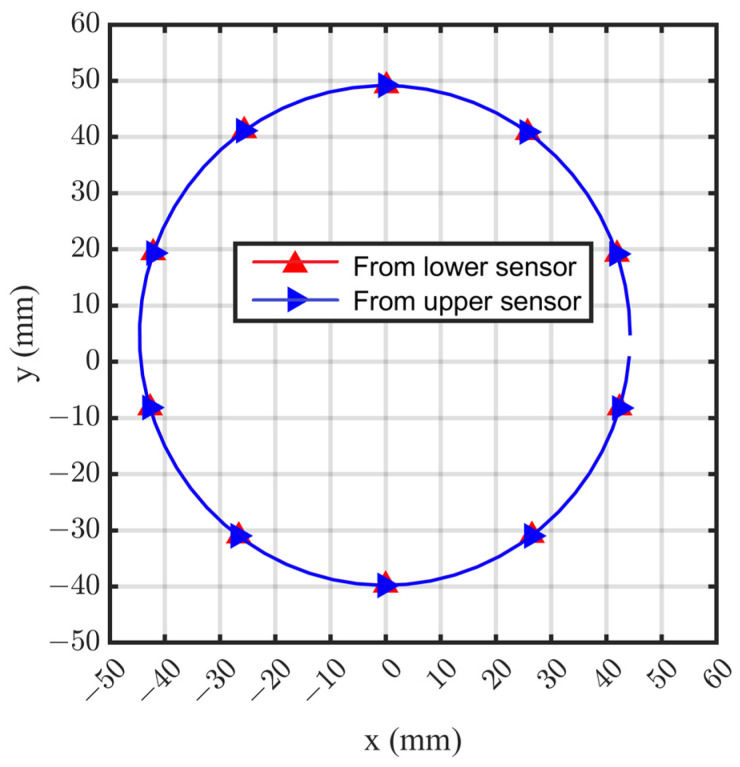
Contour points and fitting circle of the cross-section.

Then, the coordinates of 10 contour points with a 36° interval were calculated, and the cross-section of the circle can be fitted by the LSM, as shown in Figure 9. It can be seen that the calculated circle center *O_c_* did not coincide with the coordinate origin *O_d_* defined by the axis of the guide bar. Through iterative optimization, the coordinate of contour points obtained by the upper and lower laser displacement sensors were almost consistent. The calculated circle centers *O_c_* are (−0.1501, 4.6799) and (−0.1498, 4.6798), respectively, and the diameters are 88.8998 mm and 88.9488 mm, respectively. The two fitting circles were essentially superposed.

**Figure 10:**
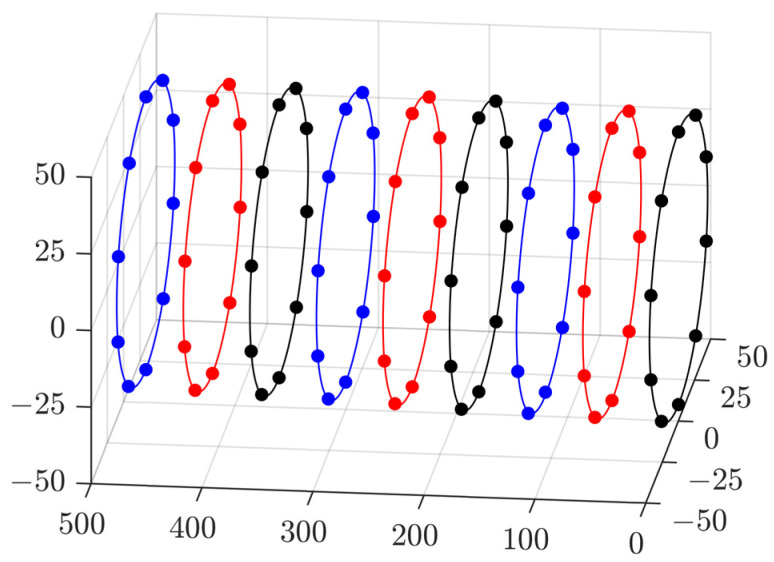
Contour points of the deep-hole inertial profile.

For each measurement process, by repeating the GDM optimization on the nine interval circular cross-sections, the point cloud coordinate information of the inertial profile of the deep-hole part can be obtained. For instance, the whole measured points at one time are shown in Figure 10.

**Figure 11:**
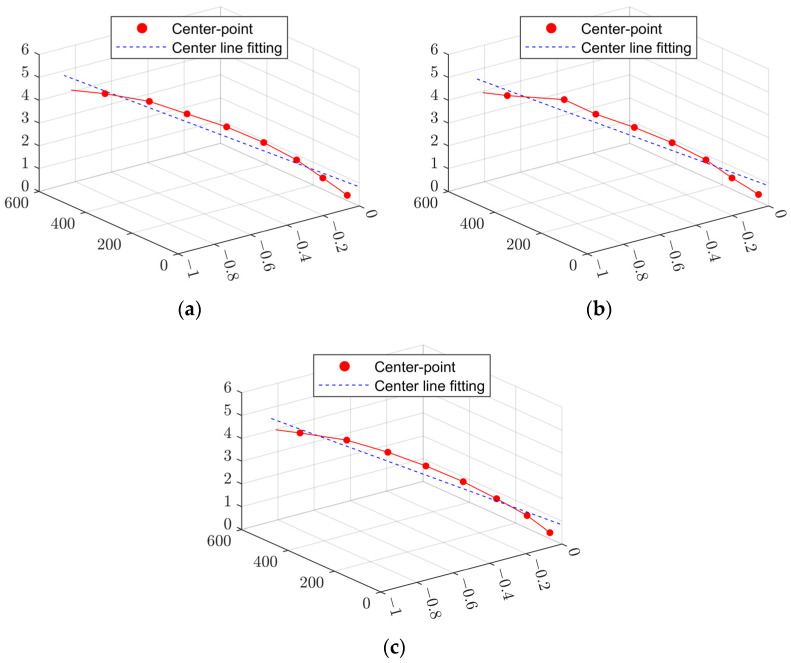
LSM fits of the center lines of the deep-hole part No. 1 for measurements at 3 times. (**a**) First measurement result; (**b**) second measurement result; and (**c**) third measurement result.

Based on the above assessment method, the roundness error of part No. 1 can be calculated, and the measurement results obtained at three times were 0.0609 mm, 0.055 mm, and 0.0615 mm. Then, the center axes were fitted for the measurements at three times, as shown in Figure 11, the evaluated results of the straightness error are about 0.2869 mm, 0.2868 mm, and 0.2873 mm, and the results exhibit good uniformity.

**Figure 12:**
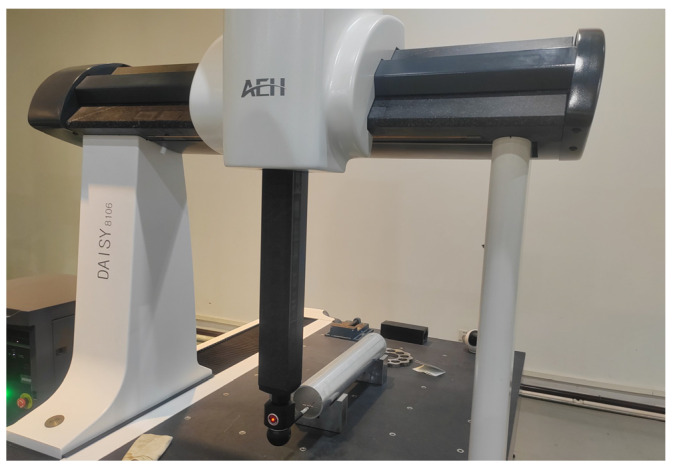
Measurement conducted by a CMM for comparison.

To verify the effectiveness of the measurement results, the deep-hole part is detected by a CMM, as shown in Figure 12. However, the measurable depth range of hole in this CMM is limited. An extension rod with a 100 mm length is required to stretch the probe into the hole, so that from the two ends, only a 200 mm range can be measured for the 500 mm parts. In contrast, the measurable range of the proposed system can reach the whole part.

The assessment error results from the proposed system and CMM are compared, as shown in Table 2. In general, the difference in the measured results between the proposed system and the CMM is inapparent, the difference in the roundness error is less than 0.01 mm, and the difference in the straightness error is less than 0.008 mm. The measured values of the CMM are lower than those of the proposed system, suggesting that maybe because the measurement range of the CMM does not cover the whole part, the error in the axial middle of the hole is unknown. Therefore, compared with the CMM, the proposed system exhibits favorable depth range and reliable precision for deep-hole measurements.

In the same way, the other four deep-hole parts were measured in sequence, and the profile accuracy was assessed, as shown in Table 3. The profile errors are discussed. Part No. 2 exhibits the smallest roundness error of 0.0578 mm, and part No. 4 has the largest error of 0.1026 mm. Part No. 1 has the smallest straightness error of 0.1772 mm, and part No. 2 has the largest error of 0.3999 mm. Part No. 4 exhibits the smallest cylindricity error of 0.6095 mm, and part No. 2 exhibits the largest error of 0.956 mm. In summary, the interior profile accuracy of these parts from the same batch indicates good consistency.

## 5. Assessment Error Analysis

Based on the experimental study, the existing errors were analyzed. According to the generating source, they can be classified as sensor error, surface finish error, positioning error of measured point, assessment method error, etc.

### 5.1. Sensor Error

The sensor errors directly affect the measurement accuracy of deep-hole parts. A high-precision, stable, and anti-disturbance displacement sensor is essential. The laser displacement sensors employed in the experiment exhibit a measurement resolution of 1 µm and a repeat positioning accuracy of 20 µm. Additionally, with the RS485 differential signal serial transmission, the external electromagnetic disturbance can be effectively shielded.

### 5.2. Surface Finish Error

The roughness and cleanliness of the interior surface of deep-hole parts also directly impact the measurement accuracy. Burrs, dust, and oil stains should be removed before the measurement process.

### 5.3. Positioning Error of Measured Points

**Figure 13:**
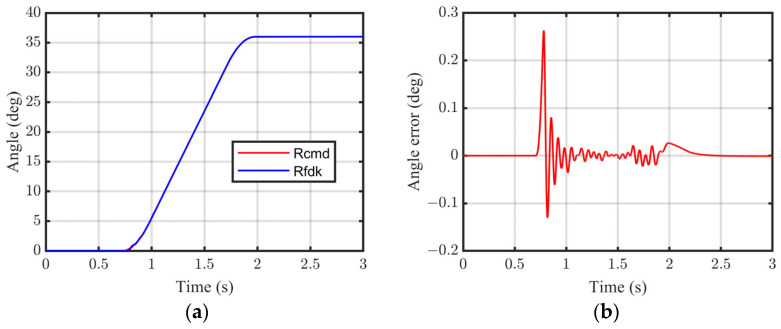
Servo motor rotating position performance. (**a**) Angle tracking and (**b**) tracking error.

**Figure 14:**
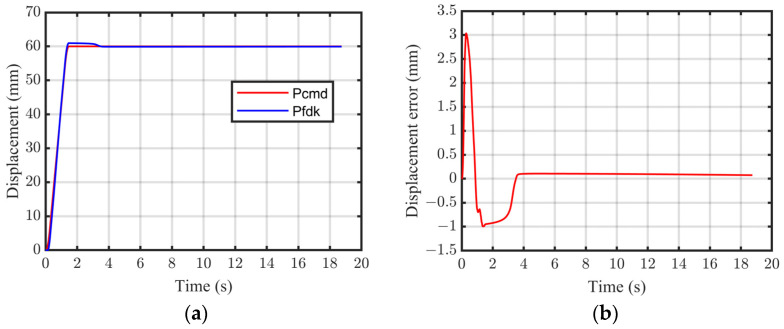
FSPMLM feed positioning performance. (**a**) Feeding position tracking and (**b**) feeding error.

The interior profile is determined by the measured point cloud information on a series of cross-sections with equal intervals, and so the positioning accuracy of the measured points significantly affects the contour geometrical precision. Combined with the 23-bit single-turn absolute encoder and 1 μm resolution grating encoder, highly precise positioning accuracy can be guaranteed by the proposed servo drive measurement system. On each cross-section, the part is rotated at an interval of 36°, and each interval is completed in 3 s; the tracking and positioning performance is shown in Figure 13. In can be seen that although the dynamic tracking error is over 0.2° in 2 s, after the positioning control in the rest time, the stable error of the angle positioning can be controlled at less than 0.01°. Furthermore, the interval of 60 mm between adjacent cross-sections is fed by the FSPMLM in each 20 s, and the tracking and positioning performance is shown in Figure 14. Controlled by the proposed system, although the dynamic tracking error is over 3 mm, the stable positioning error is less than 0.05 mm. Overall, sufficient low rotating and feeding stable errors co-guarantee the positioning accuracy of the measured points.

### 5.4. Assessment Method Error

The roundness is calculated by the LSM to fit the actual circular cross-section with finite measured points, for which fitting error is unavoidable. Similarly, errors are also generated from the GDM optimization of the circle center estimate and the center axis fit by LSM with finite evaluated center points for straightness and cylindricity assessments. An increase in the density of measured points by shortening the rotating and feeding interval is considered; meanwhile, multiple measurements are also required. However, the computation will become complex and time-consuming, and the probability of distortion points may increase. So, a comprehensive discussion of the assessment method error will be carried out in further research.

## 6. Conclusions

A servo drive interior profile measurement system for deep-hole parts is developed. The rotation of the deep-hole part is driven by the servo motor with a rotational positioning error of 0.01°, and the axial feeding of the measuring sensor is driven by the FSPMLM with a feed positioning error less than 0.05 mm. On this basis, an assessment method for the roundness error, the straightness error, and the cylindricity error of the deep-hole parts are proposed. Considering the position deviation among the source of the laser sensor, the part center, and the part rotating center, the GDM is employed to process the data from the upper and lower laser displacement sensors. The error target value of the fitting coordinate of the circle center and diameter is set to less than 0.001. A prototype of the measuring device was built for the experimental study. Compared to the CMM results, the difference in the roundness error is less than 0.01 mm, and the difference in the straightness error is less than 0.008 mm. The results show that the proposed measuring device and assessment method exhibit an effective and feasible deep-hole measurable range and reliable measure precision. Furthermore, regardless of the measurement method used, various intermediate parameters in the measuring process will bring about errors. In future work, further research on the uncertainty of deep-hole profile measurements will be conducted, along with improvements in the hardware and algorithm optimization.

## Figures and Tables

**Table 1:** Data acquired by the two laser sensors.

Laser Sensor	Point 1 (mm)	Point 2 (mm)	Point 3 (mm)	Point 4 (mm)	Point 5 (mm)	Point 6 (mm)	Point 7 (mm)	Point 8 (mm)	Point 9 (mm)	Point 10 (mm)
Sensor 1	39.71	39.33	38.79	38.23	37.89	37.93	38.37	38.91	39.47	39.75
Sensor 2	49.26	49.71	50.31	50.86	51.08	50.95	50.49	49.77	49.44	49.21

**Table 2:** Comparison of assessment error results between the proposed system and CMM.

Measurement No.	Roundness Error (mm)	Straightness Error (mm)	Cylindricity Error (mm)
1st measurement with the proposed system	0.0609	0.2869	1.0097
2nd measurement with the proposed system	0.055	0.2868	0.924
3rd measurement with the proposed system	0.0615	0.2873	0.916
1st measurement with the CMM	0.0519	0.206	NCD

**Table 3:** Assessment error results for the five deep-hole parts.

Part Number	Roundness Error (mm)	Straightness Error (mm)	Cylindricity Error (mm)
No. 1	0.0615	0.2873	0.916
No. 2	0.0578	0.3999	0.956
No. 3	0.0708	0.2001	0.7548
No. 4	0.1026	0.2616	0.6095
No. 5	0.0713	0.1772	0.6543

## Data Availability

The original contributions presented in the study are included in the article, further inquiries can be directed to the corresponding author.

## Appendix A

**Algorithm A1. Circular-section parameter calculation based on LSM with GDM**
%%Matlab program for rotating axial error compensation based on the gradient descent method, as follows: %%

function [p] = Circ_grad(ya,yb)
N = size(ya,2);
xr = sym(‘xr’);
yr = sym(‘yr’);

for i = 1:1:N
xna(i) = (0-xr)*cos((N-i)*2*pi/N)-(ya(i)-yr)*sin((N-i)*2*pi/N) + xr;
yna(i) = (0-xr)*sin((N-i)*2*pi/N)+(ya(i)-yr)*cos((N-i)*2*pi/N) + yr;
xnb(i) = (0-xr)*cos((N-i)*2*pi/N)-(yb(i)-yr)*sin((N-i)*2*pi/N) + xr;
ynb(i) = (0-xr)*sin((N-i)*2*pi/N)+(yb(i)-yr)*cos((N-i)*2*pi/N) + yr;
end

sum_xa = 0;
sum_ya = 0;
sum_xxa = 0;
sum_yya = 0;
sum_xxxa = 0;
sum_yyya = 0;
sum_xya = 0;
sum_xyya = 0;
sum_xxya = 0;
sum_xb = 0;
sum_yb = 0;
sum_xxb = 0;
sum_yyb = 0;
sum_xxxb = 0;
sum_yyyb = 0;
sum_xyb = 0;
sum_xyyb = 0;
sum_xxyb = 0;

for i = 1:1:N
sum_xa = sum_xa + xna(i);
sum_ya = sum_ya + yna(i);
sum_xxa = sum_xxa + xna(i) * xna(i);
sum_yya = sum_yya + yna(i) * yna(i);
sum_xxxa = sum_xxxa + xna(i) * xna(i) * xna(i);
sum_yyya = sum_yyya + yna(i) * yna(i) * yna(i);
sum_xya = sum_xya + xna(i) * yna(i);
sum_xxya = sum_xxya + xna(i) * xna(i) * yna(i);
sum_xyya = sum_xyya + xna(i) * yna(i) * yna(i);
sum_xb = sum_xb + xnb(i);
sum_yb = sum_yb + ynb(i);
sum_xxb = sum_xxb + xnb(i) * xnb(i);
sum_yyb = sum_yyb + ynb(i) * ynb(i);
sum_xxxb = sum_xxxb + xnb(i) * xnb(i) * xnb(i);
sum_yyyb = sum_yyyb + ynb(i) * ynb(i) * ynb(i);
sum_xyb = sum_xyb + xnb(i) * ynb(i);
sum_xxyb = sum_xxyb + xnb(i) * xnb(i) * ynb(i);
sum_xyyb = sum_xyyb + xnb(i) * ynb(i) * ynb(i);
end

Da = N * sum_xya - sum_xa * sum_ya;
Ca = N * sum_xxa - sum_xa * sum_xa;
Ea = N * sum_xxxa + N*sum_xyya -(sum_xxa + sum_yya) * sum_xa;
Ga = N * sum_yya - sum_ya * sum_ya;
Ha = N * sum_yyya + N * sum_xxya - (sum_xxa + sum_yya) * sum_ya;

Db = N * sum_xyb - sum_xb * sum_yb;
Cb = N * sum_xxb - sum_xb * sum_xb;
Eb = N * sum_xxxb + N*sum_xyyb -(sum_xxb + sum_yyb) * sum_xb;
Gb = N * sum_yyb - sum_yb * sum_yb;
Hb = N * sum_yyyb + N * sum_xxyb - (sum_xxb + sum_yyb) * sum_yb;

aa = (Ha*Da-Ea*Ga)/(Ca*Ga-Da*Da);
ba = (Ha*Ca-Ea*Da)/(Da*Da-Ga*Ca);
ca = -((sum_xxa + sum_yya) + aa * sum_xa + ba * sum_ya)/N;
xca = -0.5*aa;
yca = -0.5*ba;
Ra = 0.5 * sqrt(aa*aa+ba*ba-4*ca);

ab = (Hb*Db-Eb*Gb)/(Cb*Gb-Db*Db);
bb = (Hb*Cb-Eb*Db)/(Db*Db-Gb*Cb);
cb = -((sum_xxb + sum_yyb) + ab * sum_xb + bb * sum_yb)/N;
xcb = -0.5*ab;
ycb = -0.5*bb;
Rb = 0.5 * sqrt(ab*ab+bb*bb-4*cb);

Fa = (xca-xcb)^2+(yca-ycb)^2;
df = gradient(Fa,[xr,yr]);

xr = 0;
yr = 0;
FAA = eval(Fa);
dff = eval(df);
d = 100;
M = 100;
while (FAA > 0.0001)&&(M > 0)
xr = xr-dff(1)*d;
yr = yr-dff(2)*d;
FAA = eval(Fa);
dff = eval(df);
M = M-1;
end

p(1) = xr;
p(2) = yr;
p(3) = FAA;
p(4) = eval(xca);
p(5) = eval(yca);
p(6) = eval(xcb);
p(7) = eval(ycb);
p(8) = eval(Ra);
p(9) = eval(Rb);
p(10) = M;
%%END%%.
